# High-performance flexible perovskite solar cells exploiting Zn_2_SnO_4_ prepared in solution below 100 °C

**DOI:** 10.1038/ncomms8410

**Published:** 2015-06-22

**Authors:** Seong Sik Shin, Woon Seok Yang, Jun Hong Noh, Jae Ho Suk, Nam Joong Jeon, Jong Hoon Park, Ju Seong Kim, Won Mo Seong, Sang Il Seok

**Affiliations:** 1 Division of Advanced Materials, Korea Research Institute of Chemical Technology, 141 Gajeong-Ro, Daejeon 305-600, Korea; 2 Department of Materials Science and Engineering, Seoul National University, Seoul 151-744, Korea; 3 WCU Hybrid Materials Program, Seoul National University, Seoul 151-744, Korea; 4 Department of Energy Science, 2066 Seoburo, Jangan-gu, Sungkyunkwan University, Suwon 440-746, Republic of Korea

## Abstract

Fabricating inorganic–organic hybrid perovskite solar cells (PSCs) on plastic substrates broadens their scope for implementation in real systems by imparting portability, conformability and allowing high-throughput production, which is necessary for lowering costs. Here we report a new route to prepare highly dispersed Zn_2_SnO_4_ (ZSO) nanoparticles at low-temperature (<100 °C) for the development of high-performance flexible PSCs. The introduction of the ZSO film significantly improves transmittance of flexible polyethylene naphthalate/indium-doped tin oxide (PEN/ITO)-coated substrate from ∼75 to ∼90% over the entire range of wavelengths. The best performing flexible PSC, based on the ZSO and CH_3_NH_3_PbI_3_ layer, exhibits steady-state power conversion efficiency (PCE) of 14.85% under AM 1.5G 100 mW·cm^−2^ illumination. This renders ZSO a promising candidate as electron-conducting electrode for the highly efficient flexible PSC applications.

## Abstract (web summary)

There has been impressive progress in the development of perovskite solar cells in recent years, but the best performing systems tend to be fabricated on glass surfaces. Here, the authors present a cell built on a polymer substrate, allowing flexibility whilst maintaining high efficiency.

## Graphical abstract

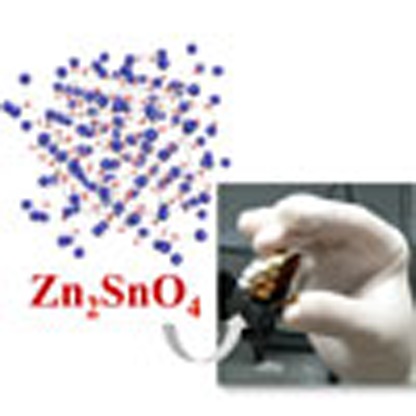

Since the first application of organic–inorganic perovskite materials into solar cells in 2009 (ref. 1), tremendous progress has been made in this field. Recently, we have demonstrated the confirmed efficiencies exceeding 18% for small-sized devices^2^. Highly efficient perovskite solar cells (PSCs) with ignorable hysteresis mainly use mesoporous (mp)-TiO_2_ as the electron acceptor and hole barrier layer^3^,^4^,^5^, although several materials, including Al_2_O_3_ (ref. 6), ZrO_2_ (ref. 7) and SrTiO_3_ (ref. 8) and so on, have been applied as electrodes. The drawback with mp-TiO_2_ is that a high-temperature process (>400 °C) is required, which prevents the use of low-cost, lightweight and flexible plastic substrates as they are unstable at high temperatures. Hence, low-temperature processable metal oxides are required for the construction of industrial printing processes with high-throughput production lines, in order to achieve the associated potential reduction in manufacturing costs^9^,^10^. In addition, the use of plastic substrates can enable portable, conformable and lightweight solar cells linked with consumer electronics. However, the PCEs of flexible solar cells fabricated on plastic substrates have generally been very low in comparison with those of PSCs fabricated on rigid substrates.

The first PSC using low-temperature processed metal oxide on a flexible substrate was demonstrated with a very low PCE of 2.62% by Mathews's group using ZnO nanorods^11^. More recently, PCE of 10.2% was obtained using ZnO nanoparticles (NPs) deposited on polymer substrates, whereas low-temperature processed mp-TiO_2_ delivered solar cells with 8.4% efficiency^12^. Most recently, Jung *et. al*.^13^ reported an efficient flexible PSC exhibiting champion PCE of 12.2% using a metal oxide electron transport layer; however, they used a compact TiO_*x*_ layer, deposited by atomic layer deposition, which is not solution processable. This state-of-the-art flexible PSC has provoked our interest in the use of other solution-processable metal oxide to further improve the performance. However, the synthesis of crystalline metal oxide NPs in solution requires mostly high temperature and high pressure. Furthermore, it will be important to prepare the particles which have capabilities to form a uniform and dense layer without additional steps, in particular at elevated temperature.

Zn_2_SnO_4_ (ZSO) is well known as a transport-conducting oxide for use in optoelectronic applications, due to its acceptable electrical and optical properties^12^. It is an n-type semiconductor with a small electron effective mass of 0.23 m_e_ and a high-electron Hall mobility of 10–30 cm^2^ Vs^−1^ (ref. 14). In addition, it has a wide optical band gap of 3.8 eV and a relatively low refractive index of ∼2.0 in the visible spectrum^15^,^16^. Furthermore, the conduction band edge position that is similar to that of TiO_2_ and ZnO makes it an excellent photoelectrode in emerging solar cell technologies, such as PSC, dye sensitized solar cell (DSSC) and organic photovoltaic (OPV)^17^,^18^,^19^. Finally, the most attractive attribute of crystalline ZSO is its chemical stability with respect to acid/base solution and polar organic solvents, for solution processing^20^,^21^. However, the ZSO ternary system is not easy to synthesize as highly dispersed NPs, and requires rather high temperatures (>200 ^o^C) to crystallize, compared with binary oxide systems (that is, TiO_2_, ZnO and SnO_2_) because both Zn and Sn ions must be regulated during a synthetic reaction. Generally, the synthesis temperature of ZSO is considerably influenced by the type of zinc precursor complex^19^,^22^. In the conventional route, ZSO NPs are synthesized with a strong base, such as NaOH, via ZnSn(OH)_6_ intermediate phase. However, a high reaction temperature (>200 °C) is required for the transformation of ZnSn(OH)_6_ into crystalline ZSO^23^. Several groups have attempted to reduce the reaction temperature by controlling the Zn complex-precursors using -amine and -carbonate mineralizers, resulting in the formation of Zn(OH)_4_^2−^ and Zn_5_(CO_3_)_2_(OH)_6_ (refs 19, 22, 24). However, high temperatures (>150 °C) are still required for the dissociation/condensation process with Sn(OH)_6_^2−^, inducing irregular shaped and agglomerated ZSO NPs.

Here, we report flexible PSCs comprised of a polyethylene naphthalate/indium-doped tin oxide (PEN/ITO) substrate, a low-temperature solution-processed ZSO layer, a CH_3_NH_3_PbI_3_ perovskite layer, a poly(triarylamine) hole conductor layer and Au as the electrode. Notably, the presence of the ZSO layer allowed superior transmittance in the visible regions, compared with bare flexible PEN/ITO substrate. This was due to an anti-reflection effect, attributable to the low refractive index of ∼1.37. A high PCE, exceeding 15%, with high-quantum efficiency was achieved. To the best of our knowledge, this PCE is the highest performance reported for flexible PSCs using metal oxide electrodes^11^,^13^,^25^.

## Results

### Synthesis of ZSO NPs via Zn-hydrazine complex precursor

**Figure 1:**
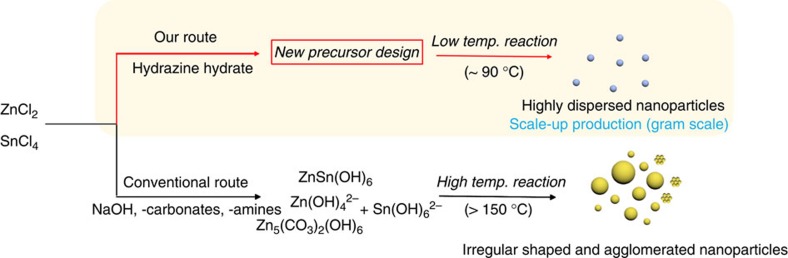
Synthetic procedure for ZSO. Schematic illustration for the formation of highly dispersed ZSO NPs with a reaction temperature below 100 °C.

**Figure 2:**
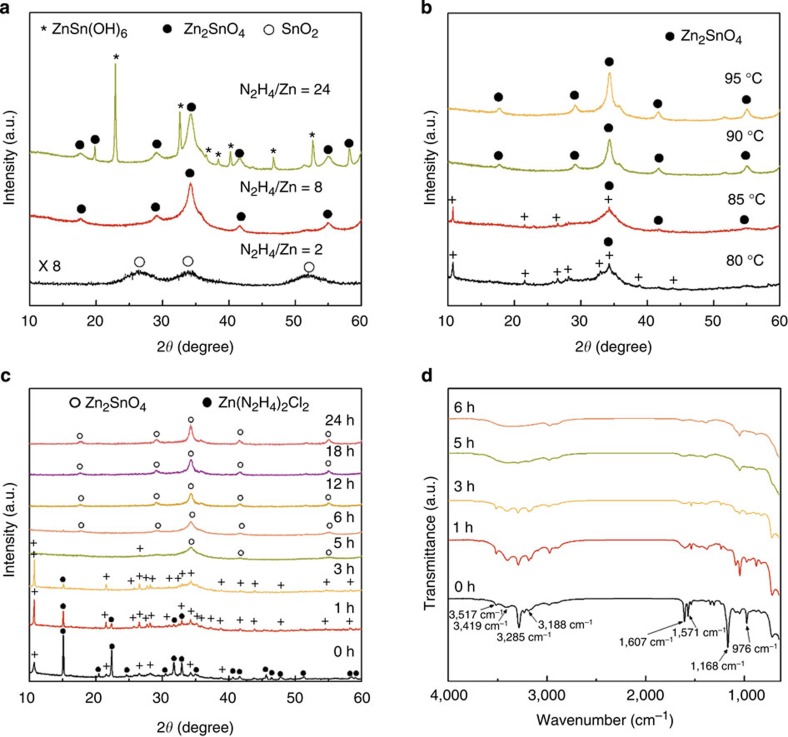
XRD and Fourier transform infrared (FT-IR) of samples obtained with various reaction conditions. Powder X-ray diffractograms (CuKα radiation) of (**a**) different N_2_H_4_/Zn ratios (90 °C, 12 h), (**b**) different reaction temperatures (N_2_H_4_/Zn=8, 12 h) and (**c**) different reaction times (90 °C, N_2_H_4_/Zn=8). (**d**) FT-IR spectra of samples synthesized at different reaction times (90 °C, N_2_H_4_/Zn=8). Unindexable peaks are denoted as (+).

The strategy for synthesizing ZSO NPs below 100 °C is schematically depicted in Fig. 1. To synthesize highly dispersed, low-temperature ZSO NPs, the chemical reactions were carried out at 90 °C for 12 h, with various molar N_2_H_4_/Zn ratios. Figure 2a shows the X-ray diffraction (XRD) patterns of powders synthesized at 90 °C for 12 h, as a function of the different molar N_2_H_4_/Zn ratios (2, 8 and 24). At a low N_2_H_4_ concentration (N_2_H_4_/Zn=2), the only pure SnO_2_ phase is observed, whereas a pure ZSO phase with an inverse spinel structure (JCPDS 24-1470) is obtained at N_2_H_4_/Zn ratio=8. However, an excessive of N_2_H_4_, that is, N_2_H_4_/Zn=24, produces highly crystalline ZnSn(OH)_6_ as a secondary phase besides ZSO. It is well known that N_2_H_4_ can act, not only as a complexing agent but also as an OH supplier by a dissociation reaction during the reaction process^26^,^27^. Therefore, the variance in OH concentration caused by N_2_H_4_ can influence the formation of crystalline phases (SnO_2_, ZSO and ZnSn(OH)_6_). Figure 2b shows XRD patterns of the synthesized powders with different reaction temperature, from 80 to 95 ^o^C, with N_2_H_4_/Zn=8. Unindexable peaks, denoted by ‘+', are observed with the ZSO phase at 80 and 85 ^o^C, whereas all peaks are indexed by the ZSO phase at 90 and 95 ^o^C. The unindexable peaks (to be discussed later) are related to new Zn–N–H–OH complexes in this process, indicating that a temperature below 90 ^o^C is insufficient to drive the dissociation/condensation reaction of such complex precursor for the formation of ZSO crystal. Therefore, N_2_H_4_ concentration and reaction temperature are the key factors for synthesizing pure crystalline ZSO at a temperature below 100 °C. To understand the formation mechanism of ZSO crystals by N_2_H_4_ complexing below 100 °C, a time-dependent experiment was performed under certain conditions (N_2_H_4_/Zn=8 and 90 °C). Figure 2c shows the XRD patterns of powder prepared at different reaction times. According to the XRD traces, before heating (0 h), peaks (●) indexed for Zn(N_2_H_4_)_2_Cl_2_ (JCPDS 72-0620) are dominant compared with the unindexable peaks (+). However, the unindexable peaks predominate over the peaks corresponding to Zn(N_2_H_4_)_2_Cl_2_ after 1 h of heating. The peaks corresponding to Zn(N_2_H_4_)_2_Cl_2_ gradually disappear, whereas unindexble peaks, including the main peak at 10.7 °, further develop with an increase in the reaction time until 3 h, implying the transformation of Zn(N_2_H_4_)_2_Cl_2_ into new complex. After 5 h, most of the peaks based on the new complex disappear and pure ZSO crystal phases are observed with complete decomposition of the new complex. Also, the intensity of the ZSO peaks increase with an increase in the reaction time (12, 18 and 24 h), suggesting an increase in particle size (Supplementary Fig. 1). Our process was summarized in a flow chart (Supplementary Fig. 2). Based on these results, it can be concluded that the formation of new complex-precursors has a decisive effect on the low-temperature formation of ZSO crystals.

Fourier transform infrared analysis was performed to reveal the possible composition of the new complex-precursors and to further study the formation mechanism (Fig. 2d). At 0 h, the characteristic peaks of Zn(N_2_H_4_)_2_Cl_2_, such as N–H stretching (3,188 and 3,285 cm^−1^), NH_2_ bending (1,571 and 1,607 cm^−1^), NH_2_ twisting (1,168 cm^−1^) vibration and N–N stretching (976 cm^−1^) vibration are observed and are accordant with the reported values^28^. In particular, the peak at 976 cm^−1^ appears when the N_2_H_4_ coordinates two metal ions in a bidentate bridging mode, which is strong evidence for the formation of a metal hydrazine complex^29^. Importantly, with an increase in reaction time up to 3 h at 90 °C, the N–N stretching (976 cm^−1^), the NH_2_ bending (1,571 and 1,607 cm^−1^) and the NH_2_ twisting (1,168 cm^−1^) peaks gradually disappear. Conversely, the N–H stretching peaks at 3,188 and 3,285 cm^−1^ remain, but with small shift, and the peaks at 3,418 and 3,516 cm^−1^ are much better pronounced. The peak at 3,418 cm^−1^ is due to stretching of the OH linked to the matrix, and the peak at 3,516 cm^−1^ is due to coordinated/adsorbed water molecules, respectively^30^. These results may imply decomposition of the zinc bishydrazine complex into a zinc ammine complex that includes OH^-^ ions (Zn–N–H–OH). In addition, by energy dispersive spectroscopy analysis, we observe the reduction and removal of Cl^-^ with the increase in reaction time from 0 to 3 h (Supplementary Fig. 3). These results indicate that new Zn–N–H–OH complex-phases are formed during the reaction, by decomposition of N_2_H_4_, removal of Cl^−^ ions, and incorporation of OH^−^ ions from initial Zn(N_2_H_4_)_2_Cl_2_ complex. As the reaction progresses over 6 h, all of the characteristic peaks for the Zn–N–H–OH complex disappear, suggesting the formation of a crystalline ZSO, which is in accordance with the XRD results.

**Figure 3:**
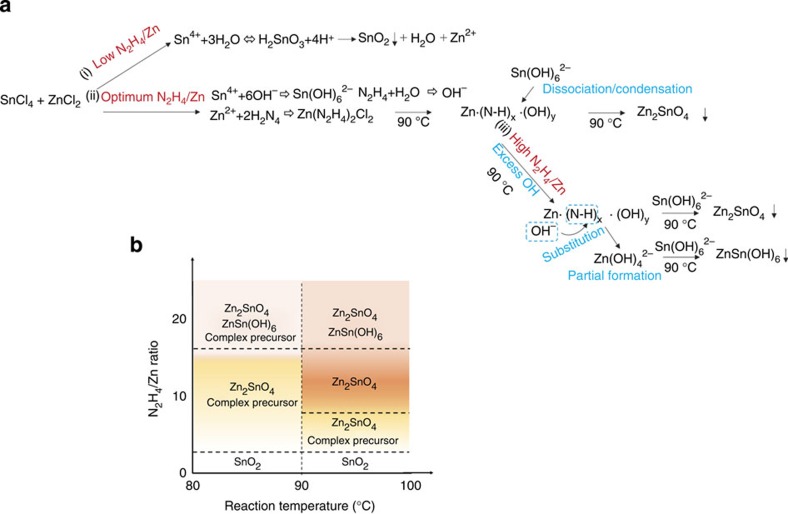
Formation mechanism of ZSO NPs. (**a**) Schematic illustration of the formation mechanism of crystalline ZSO NPs via a low-temperature process below 100** **°C. (**b**) Formation map of ZSO with different temperature and hydrazine/Zn ratio.

Based on the series of experiments described above, we propose a possible formation mechanism, as illustrated in Fig. 3a, to rationalize the formation of ZSO NPs at temperatures below 100 °C. When the hydrazine concentration is low ((i) route: acidic condition), H_2_SnO_3_ is produced due to the strong hydrolysis effect of Sn^4+^, which can lead to the formation of SnO_2_, whereas Zn^2+^ ions remain in the solution and are washed away after the reaction^23^. On the other hand, an appropriate concentration of hydrazine ((ii) route: mild alkaline condition) prevents the hydrolysis reaction of Sn^4+^, and favour the formation of Sn(OH)_6_^2−^ rather than H_2_SnO_3_ (ref. 21), whereas the Zn^2+^ ion forms a Zn(N_2_H_4_)_2_Cl_2_ complex with hydrazine^31^,^32^. In metal-hydrazine systems, the metal/hydrazine ratio is an important factor for determining the composition of the hydrazine complex such as M(N_2_H_4_)_1_XCl_2_, M(N_2_H_4_)_2_XCl_2_ and M(NH_3_)_*X*_Cl_2_ (refs 33, 34). In our case (N_2_H_4_/Zn=8), Zn(N_2_H_4_)_2_Cl_2_ is the dominant form initially (at 0 h), as it is more stable than other compositions. As the reaction progresses, the zinc ammine hydroxo complex, Zn–N–H–OH, is formed by the reaction of Zn(N_2_H_4_)_2_Cl_2_ with excess N_2_H_4_, and a continuous supply of OH^-^ (ref. 35). Compared with other metal complexes, the metal ammine hydroxo complex requires a relatively low temperature (<100 °C) for the formation of the crystalline metal oxide. This is due to the low-energy kinetics of metal-ammine dissociation and the hydroxide condensation/dehydration reaction^36^,^37^. Therefore, we believe that Zn–N–H–OH complexes can lead to the formation of crystalline ZSO with Sn(OH)_6_^2-^, even below 100 °C. However, an excess hydrazine ((iii) route: alkaline condition) leads to the formation of ZnSn(OH)_6_ as a secondary phase. The increased pH favours the substitution of N–H with OH^-^ in the Zn–N–H–OH complex, resulting in the partial formation of Zn(OH)_4_^2-^ (ref. 38). In this case, ZnSn(OH)_6_ can be produced as a secondary phase, in which the transformation of ZnSn(OH)_6_ into ZSO requires a high temperature (>200 °C)^23^. As a result, both ZSO and ZnSn(OH)_6_ are formed during the 90 °C reaction. From these results and additional detailed experiments, we propose a rough formation map of ZSO, which outlines the effects of variations in temperature and N_2_H_4_/Zn ratio, for the process below 100 ^o^C (Fig. 3b). As shown in the map, an N_2_H_4_/Zn ratio in the range of 8–24 at a temperature of around 90 °C, leads to a crystalline ZSO phase without secondary phases, which is the first demonstration of the synthesis of pure ZSO at a low temperature (<100 °C). This mild synthesis condition is comparable to that of binary oxides (TiO_2_ or ZnO), and can provide powerful competitiveness as an alternative to binary oxides for various device applications.

### Deposition of ZSO thin layers onto substrates

**Figure 4:**
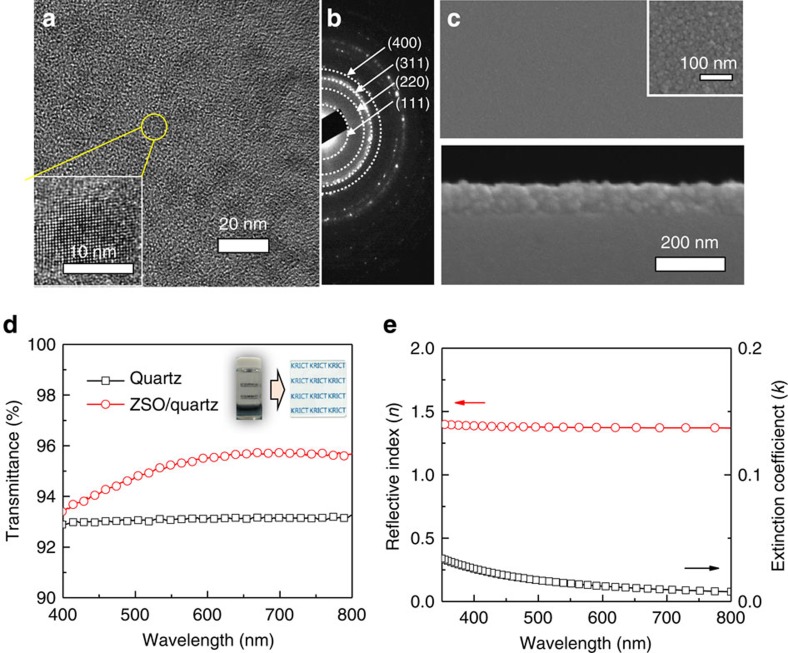
Deposition and optical properties of ZSO film. (**a**) TEM (inset: high-resolution TEM) and (**b**) SAED pattern of ZSO NPs synthesized at 90 °C for 12 h (N_2_H_4_/Zn=8). (**c**) Plane view and cross-sectional SEM image of ZSO thin film (inset in Fig. 4c: high-magnification SEM image). (**d**) transmittance spectra of ZSO films on fused silica substrate with four coating times (inset: the photograph of ZSO colloidal solution and the resultant ZSO film). (**e**) The reflective index (*n*) and the extinction coefficient (*k*) of low-temperature processed ZSO film.

Figure 4a,b shows representative transmission electron microscopy (TEM) images and selected area electron diffraction (SAED) patterns of the ZSO NPs synthesized at 90 °C for 12 h. The TEM image exhibits highly dispersed and defined particles with a narrow size distribution (∼11 nm). The formation of uniform and dispersed NPs can be ascribed to the low reaction temperature and the formation of a stable precursor complex with hydrazine^33^. In addition, the high-resolution TEM image (inset in Fig. 4a) and the SAED patterns (Fig. 4b) are in agreement with the spinel structure of ZSO deduced from XRD patterns. Moreover, elemental mapping by energy dispersive spectroscopy indicates the homogeneous distribution of Zn and Sn elements in the NPs, as presented in Supplementary Fig. 4. For fabrication of high throughput and flexible optoelectronic applications, characterization of the low-temperature, solution-processed ZSO film is required. We deposited ZSO film on fused silica substrates by spin-coating and drying at 100 °C, using the resultant crystalline ZSO colloidal solution. The scanning electron microscopy (SEM) images of the ZSO film are shown in Fig. 4c. The plane image (Fig. 4c above) reveals that the surface exhibits crack-free, uniform morphology, with densely packed NPs (inset in Fig. 4c above). The atomic force microscopy analysis (Supplementary Fig. 5) indicates the resultant ZSO film has a flat surface with a root-mean-square roughness of 2.07 nm. Moreover, the entire substrate surface is uniformly covered by ZSO NPs with a thickness of ∼100 nm (Fig. 4c below). Figure 4d shows the optical transmission spectra of a ZSO film on quartz after several coating cycles. The transmittance of ZSO films is comparable to bare fused silica substrates over the entire wavelength region, regardless of their thickness (various coating cycles, Supplementary Fig. 6). The photograph (inset in Fig. 4d) shows that the ZSO film fabricated from the highly dispersed ZSO colloidal solution is highly transparent. The high transparency can be ascribed to the optical properties of the ZSO film. Figure 4e shows the refractive index (*n*) and extinction coefficient (*k*) spectra for ∼100-nm-thick ZSO film deposited on a silicon substrate measured using spectroscopic ellipsometry. The refractive index *n* is significantly small around 1.37 throughout the entire visible range compared with the reported value of 2.0 for ZSO film^16^. Because the refractive index for NP film has been reported to be determined by its crystallinity and porosity^39^,^40^, the ZSO film prepared from the low-temperature ZSO colloidal solution may possess low refractive index. In addition, the low extinction-coefficient value of nearly 0 in the visible region is in accordance with previously reported one^16^. The unique optical properties of the ZSO film such as lower refractive index (*n*∼1.37) than SiO_2_ glass (*n*∼1.5), wide band gap, and low-extinction coefficient (*k*) are causes of improved transmittance of the fused silica substrate, even after coating with the ZSO film. These results reveal that the low-temperature-synthesized ZSO NPs can facilitate the formation of highly transparent and uniform film on substrate without additional treatment.

### Fabrication of ZSO-based flexible PSCs

**Figure 5:**
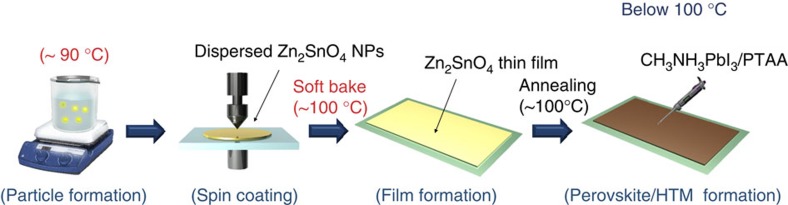
Experimental procedure for PSCs. Schematic illustration of the low-temperature process for fabricating flexible device with ZSO NPs.

**Figure 6:**
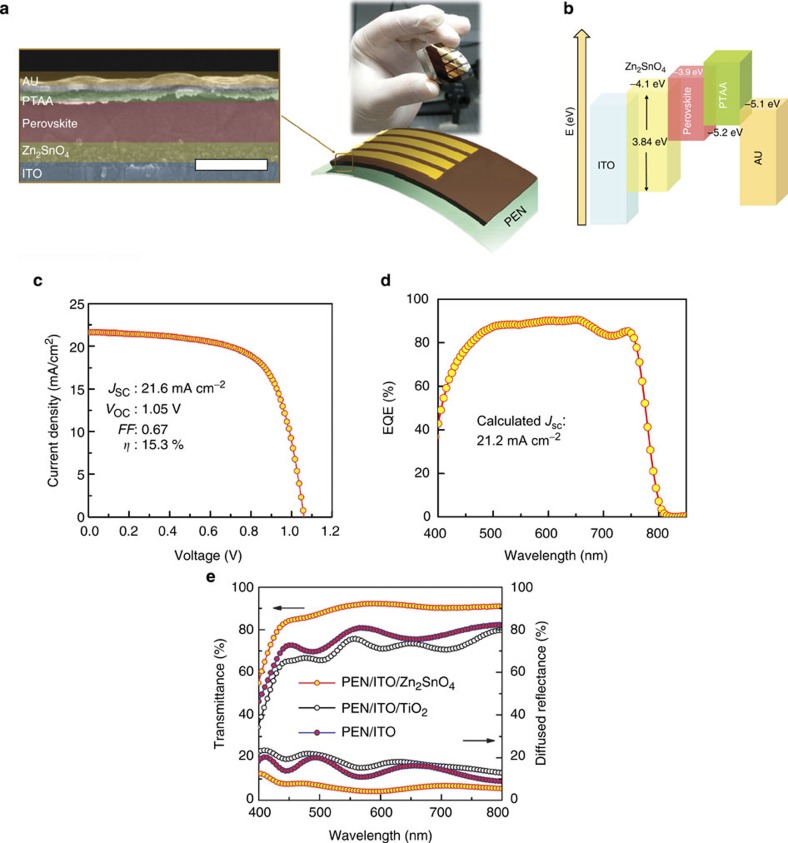
Structure and performance of ZSO-based flexible perovskite solar cell. (**a**) Cross-sectional SEM image and photograph of the ZSO-based flexible perovskite solar cell (scale bar, 500 nm). (**b**) Energy levels of the materials used in this study. (**c**) Photocurrent density–voltage (*J*–*V*) curve measured by reverse scan with 10 mV voltage steps and 40 ms delay times under AM 1.5 G illumination. (**d**) EQE spectrum of the ZSO-based flexible perovskite solar cell. (**e**) Transmittance and reflectance spectra of PEN/ITO/ZSO, PEN/ITO/TiO_2_ and PEN/ITO substrate. A dense TiO_2_ film was fabricated by TiO_2_ NPs obtained from a non-hydrolytic sol gel route^9^ (Supplementary Fig. 12).

To demonstrate its viability for high throughput and flexible optoelectronic applications, ZSO film was employed, after four coating cycles, as an electron transport layer over PEN/ITO substrate. The ZSO film on ITO substrate, in common with those seen on quartz, has flat (root-mean-square: 3.76 nm) and uniform morphology with densely packed NPs (Supplementary Fig. 7). Figure 5 shows a series of processing steps from ZSO NPs formation to flexible solar cell fabrication. All steps require very simple techniques and low processing temperature below 100 °C. This is the first demonstration of low-temperature process for a ternary oxide electron transport layer to apply in PSCs, which can possibly provide an alternative to conventional TiO_2_- or ZnO-based PSCs. Figure 6a,b presents a colour-enhanced cross-sectional SEM image of the device architecture fabricated in this study and the corresponding energy level diagram of the device based on reported values^17^,^41^, respectively. As shown in Fig. 6a, two uniform layers, ZSO layer and a perovskite layer, are observed; the flat and homogeneous ZSO film with a thickness of 110±10 nm can lead to uniform perovskite film on the ITO/ZSO substrate by using pre-reported solvent-engineering technique^3^. Furthermore, the plane-view SEM image (Supplementary Fig. 8) showed that the perovskite layer on ZSO was deposited in dense and uniformly spread film with fine-grained morphology, which is similar with our previous report^3^. The fabricated flexible PSC is shown in the photograph in Fig. 6a. Figure 6b reveals that the energy level of ZSO is similar to that of TiO_2_, implying that it has an identical working mechanism as a TiO_2_-based PSCs. Figure 6c shows the photocurrent density–voltage (*J*–*V*) curve of the ZSO-based flexible PSC under simulated solar illumination (100 mW cm^−2^ air mass (AM) 1.5G). The *J*–*V* curve exhibits a short circuit current density (*J*_sc_) of 21.6 mA cm^−2^, an open circuit voltage (*V*_oc_) of 1.05 V, and fill factor (FF) of 0.67, resulting in PCE (*η*) of 15.3% with steady-state PCE of 14.85%, which is comparable to the performance of ZSO-based PSC, prepared on ITO glass (Supplementary Fig. 9 and Supplementary Table 1). ZSO-based PSC shows the hysteresis in the *J–V* curves measured with reverse and forward scan (Supplementary Fig. 10 and Supplementary Table 2), although it is smaller than those of our TiO_2_-based planar device reported by us^3^. However, in contrast to TiO_2_-based planar PSCs reported by other groups^4^, the stabilized photocurrent density and PCE obtained from ZSO NPs-based planar PSC approaches the value measured by reverse mode (Supplementary Fig. 10). This result is consistent with that of mesosuperstructured solar cells^4^. We repeated the fabrication procedure to obtain a reliable and reproducible result. As can be seen in the histogram for the photovoltaic parameters collected from 60 independent devices (Supplementary Fig. 11 and Supplementary Table 3), around 40% of the cells show PCE over 13% with the average PCE of 13.7%. In particular, the average *J*_sc_ and *V*_oc_ exceed 20 mA cm^−2^ and 1 V, respectively, which is much greater than other flexible PSCs based on TiO_2_/ZnO/PEDOT:PSS. Furthermore, the flexible device shows a very broad external quantum efficiency (EQE) plateau over 80% between 460 and 755 nm, as shown in Fig. 6d. Integrating the product of the AM 1.5 G photon flux with the EQE spectrum yield a predicted *J*_sc_ of 21.2 mA cm^−2^, which agrees well with the measured value of 21.6 mA cm^−2^. This high EQE corresponding to a high *J*_sc_ for ZSO-based PSCs reveals that the ZSO film prepared at low-temperature produces excellent electron collecting and light harvesting ability within the device. The high performance (≥15%) of flexible PSCs is first demonstrated using the newly designed, solution-processed ZSO film, which is superior to the reported TiO_2_- and ZnO-based flexible PSCs^13^,^25^. The higher charge collecting ability might be due to the higher electron mobility of ZSO in nature than conventional TiO_2_ (refs 15, 18). In addition to the high mobility of ZSO, the lower refractive index (*n*∼1.4) of the ZSO film than TiO_2_ film (*n*∼2.5) leads to further light harvesting gain due to the anti-reflectance effect between ITO (*n*∼2.0) and ZSO or TiO_2_ layers. Figure 6e presents the transmittance and diffused reflectance spectra for bare PEN/ITO, PEN/ITO/TiO_2_ and PEN/ITO/ZSO substrate. Introduction of the ZSO film brings significant reduction of the reflectance over the entire wavelength region, resulting in improvement of transmittance from ∼75 to ∼90%. As ITO on PEN film has a lower refractive index than the ITO film on glass due to lower crystallinity by low-temperature processing, reflectance loss between ITO and TiO_2_ for flexible substrate can be larger than that of glass substrate^42^. The ZSO film overcomes the poor transmittance caused by the flexible substrate^43^, which can lead a comparable *J*_sc_ with that of a device based on glass substrate. As a result, superior electrical/optical properties of ZSO film can improve the *J*_sc_ for flexible PSCs. In addition, we performed mechanical bending tests over 300 cycles. As shown in Supplementary Fig. 13, the performance of the device retains over 95% of its initial efficiency even after 300 bending cycles.

## Discussion

A well-dispersed crystalline ZSO colloidal solution was synthesized by the introduction of a Zn–N–H–OH complex derived from hydrazine via simple solution process at 90 ^o^C. The flat and uniform ZSO films on flexible PEN/ITO substrate were also fabricated by a spin-coating method using the colloidal solution with drying at 100 ^o^C. An n-type semiconducting ZSO film with wide band gap (3.8 eV) on flexible substrate has great potential for use in flexible optoelectronic devices. The resultant ZSO film shows a very low refractive index around 1.37 throughout the entire visible range, leading to improved transmittance of PEN/ITO substrate due to anti-reflection. The PSC using the highly transparent PEN/ITO/ZSO substrate showed high-quantum efficiency and a PCE of 15.3%, which is comparable to that of device based on rigid glass. This low-temperature synthetic method can provide a breakthrough for the fabrication of metal-oxide semiconductors on flexible substrate in advanced optoelectronic applications as a technology capable of high performance and large production.

## Methods

### Synthesis of ZSO NPs

All chemicals for the preparation of NPs were of regent grade and were used without further purification. ZnCl_2_ (12.8 mmol, Aldrich) and SnCl_4_·5H_2_O (6.4 mmol, Aldrich) were dissolved in deionized water (160 ml) under vigorous magnetic stirring. N_2_H_4_·H_2_O (N_2_H_4_ molar ratio/Zn=4/1, 8/1, 24/1) was then added to the reaction solution. White precipitates formed immediately, and this solution, including the precipitate, was heated on a hot plate at 90 °C for 12 h. The obtained products were thoroughly washed with deionized water and ethanol and were then dispersed in 2-methoxy ethanol, resulting in a colloidal solution.

### Solar cell fabrication

A ZSO thin film was prepared by spin coating the colloidal dispersion of ZSO particles onto ITO-coated glass/PEN substrate at 3,000 r.p.m. for 30 s, followed by drying on a hot plate at 100 °C for 3 min. To control film thickness, the procedure was repeated four times. After baking at 100 °C for 1 h in air, the perovskite layer was deposited onto the resulting ZSO film by a consecutive two-step spin coating process at 1,000 and 5,000 r.p.m. for 10 and 20 s, respectively, from the mixture solution of methylammonium iodide (CH_3_NH_3_I) and PbI_2_. During the second spin coating step, the film was treated with toluene drop-casting, and then was dried on a hot plate at 100 °C for 10 min. The detailed preparation of the CH_3_NH_3_I has been described in previous work^3^. A solution of poly(triarylamine) (EM index, *M*_n_=17,500 g mol^−1^, 15 mg in toluene 1.5 ml) was mixed with 15 μl of a solution of lithium bistrifluoromethanesulphonimidate (170 mg) in acetonitrile (1 ml) and 7.5 μl 4-tert-butylpyridine. The resulting solution was spin coated on the CH_3_NH_3_PbI_3_/ZSO thin film at 3,000 r.p.m. for 30 s. Finally, an Au counterelectrode was deposited by thermal evaporation.

### Characterization

The crystal structure and phase of the materials were characterized using an XRD (New D8 Advance, Bruker) and SAED. The chemical composition of materials was investigated using a Fourier transform infrared spectrometer (Nicolet 6,700, Thermo Scientific). The morphologies and microstructures were investigated by field emission scanning electron microscopy (FESEM, SU 70, Hitachi), transmission electron microscopy (TEM, JEM-2,100 F, JEOL) and atomic force microscopy (NanostationII, Surface Imaging Systems). The optical properties of samples were characterized using a ultraviolet–visible spectrophotometer (UV 2,550, Shimadzu). The EQE was measured using a power source (300 W xenon lamp, 66,920, Newport) with a monochromator (Cornerstone 260, Newport) and a multimeter (Keithley 2001). The *J*–*V* curves were measured using a solar simulator (Oriel Class A, 91,195 A, Newport) with a source meter (Keithley 2,420) at 100 mW cm^−2^, AM 1.5 G illumination, and a calibrated Si-reference cell certified by the NREL. The *J–V* curves were measured by reverse scan (forward bias (1.2 V) → short circuit (0 V)) or forward scan (short circuit (0 V) → forward bias (1.2 V)). The step voltage and the delay time were fixed at 10 mV and 40 ms, respectively. The *J*–*V* curves for all devices were measured by masking the active area with a metal mask 0.096 cm^2^ in area. Time-dependent current was measured with a potentiostat (PGSTAT302N, Autolab) under one sun illumination.

## Additional information

**How to cite this article:** Shin, S. S. *et al.* High-performance flexible perovskite solar cells exploiting Zn_2_SnO_4_ prepared in solution below 100 °C. *Nat. Commun.* 6:7410 doi: 10.1038/ncomms8410 (2015).

## Supplementary Material

Supplementary Information Supplementary Figures 1-13 and Supplementary Tables 1-3
