# Supplementary material for: High-performance flexible perovskite solar cells exploiting Zn2SnO4 prepared in solution below 100 °C
Source: Nat Commun. 2015 Jun 22;6:7410. doi: 10.1038/ncomms8410 (PMC4557286; doi:10.1038/ncomms8410)
Supplement: Supplementary Information — Supplementary Figures 1-13 and Supplementary Tables 1-3 [file ncomms8410-s1.pdf]

Supplementary Figures

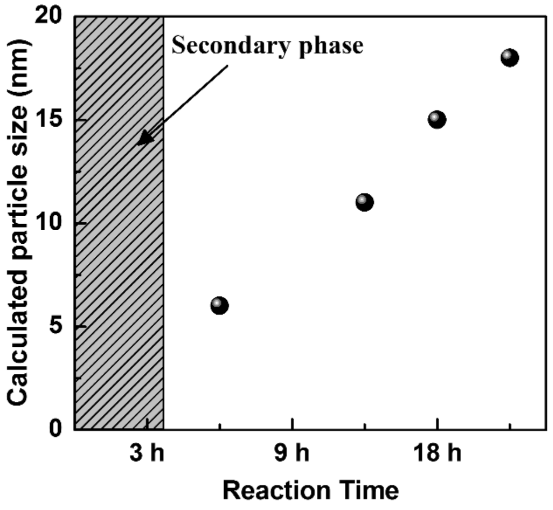

Supplementary Figure 1. The effect of reaction time on the size of ZSO NPs. The particle size was determined by scherrer's equation from XRD data.

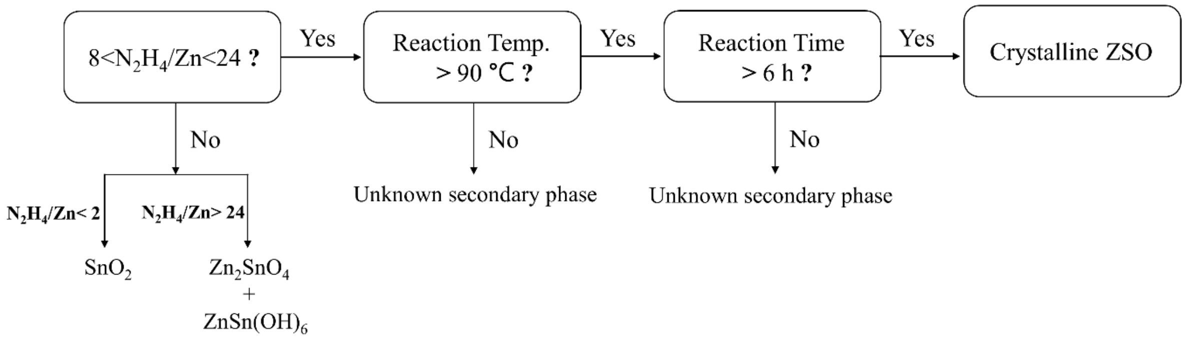

Supplementary Figure 2. Flow chart for synthesis of ZSO NPs from solution-based precursors

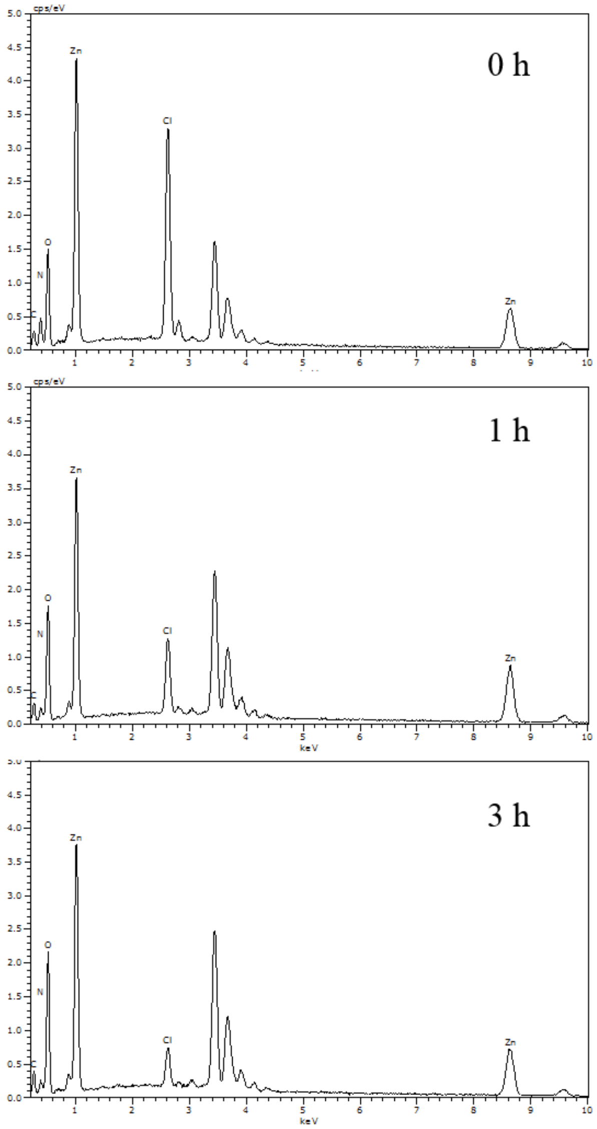

Supplementary Figure 3. Elemental analysis. EDS spectra of samples obtained with different reaction times.

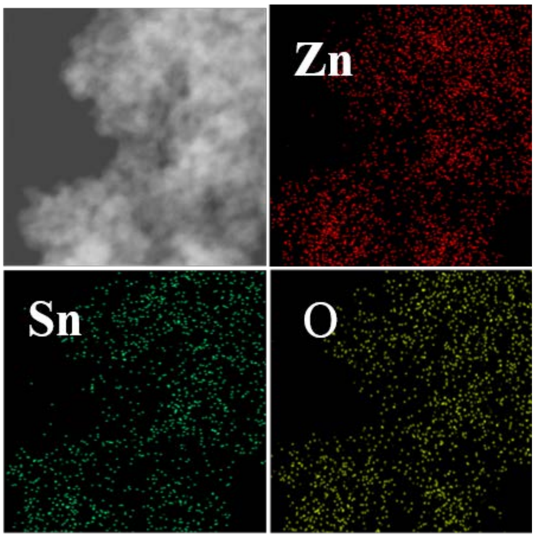

Supplementary Figure 4. Elemental analysis. EDS mapping image of ZSO NPs synthesized at 90 ˚C for 12 h (N_2H_4/Zn: 8).

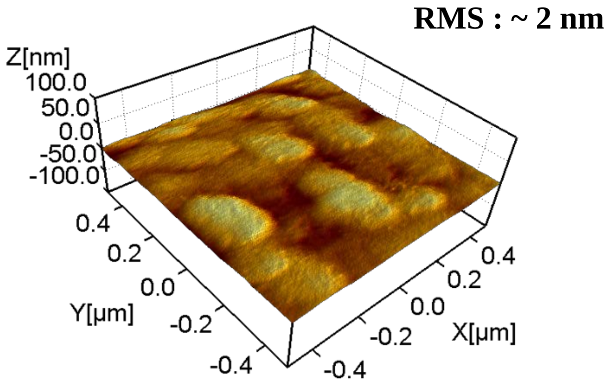

Supplementary Figure 5. Morphology of ZSO film. 3D topographical AFM image of ZSO thin film on fused silica substrate.

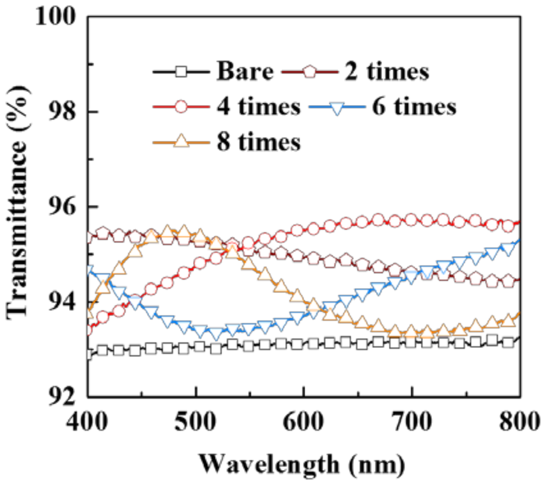

Supplementary Figure 6. Optical property of ZSO films. Transmittance spectra of ZSO films on fused silica substrate with various spin-coating times.

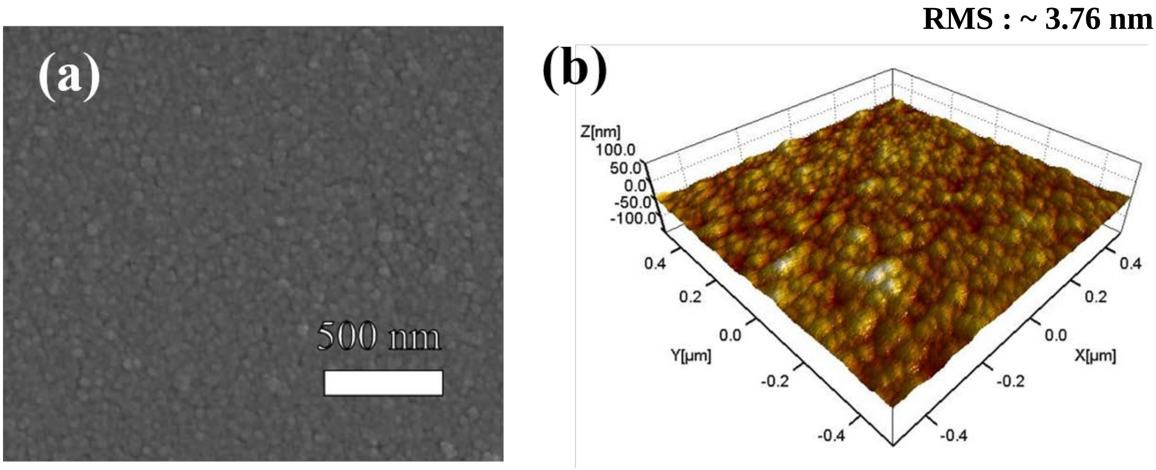

Supplementary Figure 7. Morphology of ZSO Film. (a) Plane-view SEM and (b) AFM image of ZSO film on ITO.

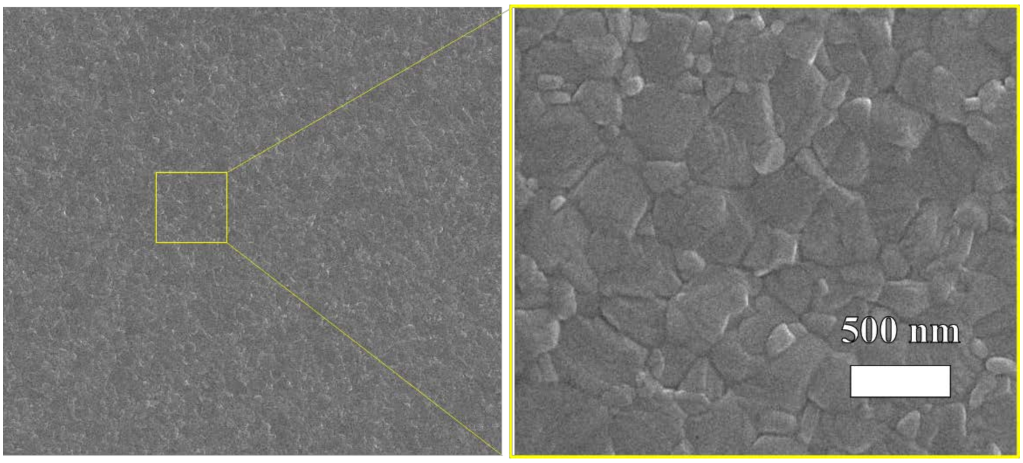

Supplementary Figure 8. Morphology of perovskite film. Plane-view SEM image of solvent-engineered perovskite layer.

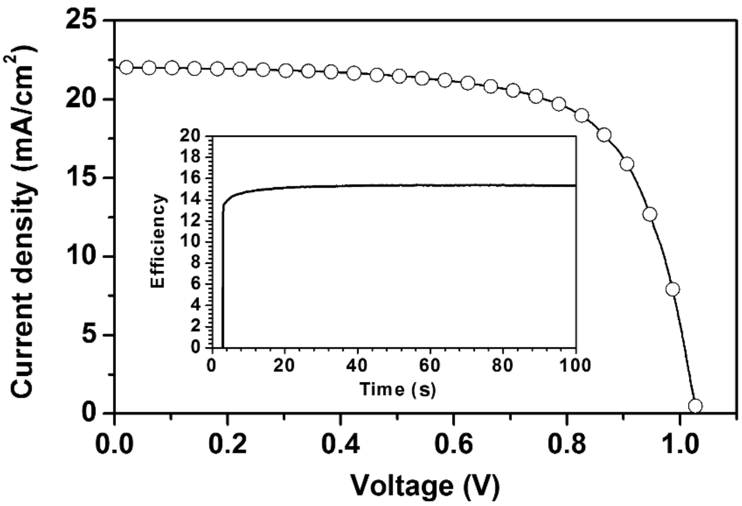

Supplementary Figure 9. Photovoltaic performance. *J*-*V* curve of the ZSO-based perovskite solar cell fabricated with an ITO glass substrate *via* all low temperature process, under AM 1.5 G illumination. The inset shows the stabilized power conversion efficiency at a maximum power point (0.83 V)

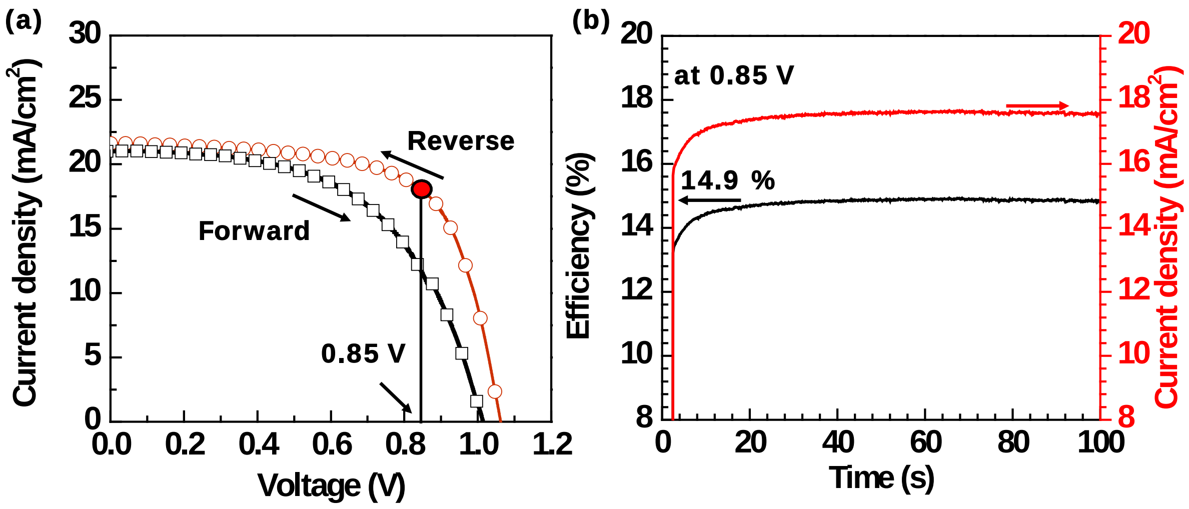

Supplementary Figure 10. Photovoltaic performance. (a) Photocurrent density-voltage (*J-V*) curves for ZSO-based flexible perovskite solar cell measured by forward and reverse scans with 10 mV voltage steps and 40 ms delay times under AM 1.5 G illumination. (b) Steady-state current measured at a maximum power point (0.85 V) and stabilized power conversion efficiency.

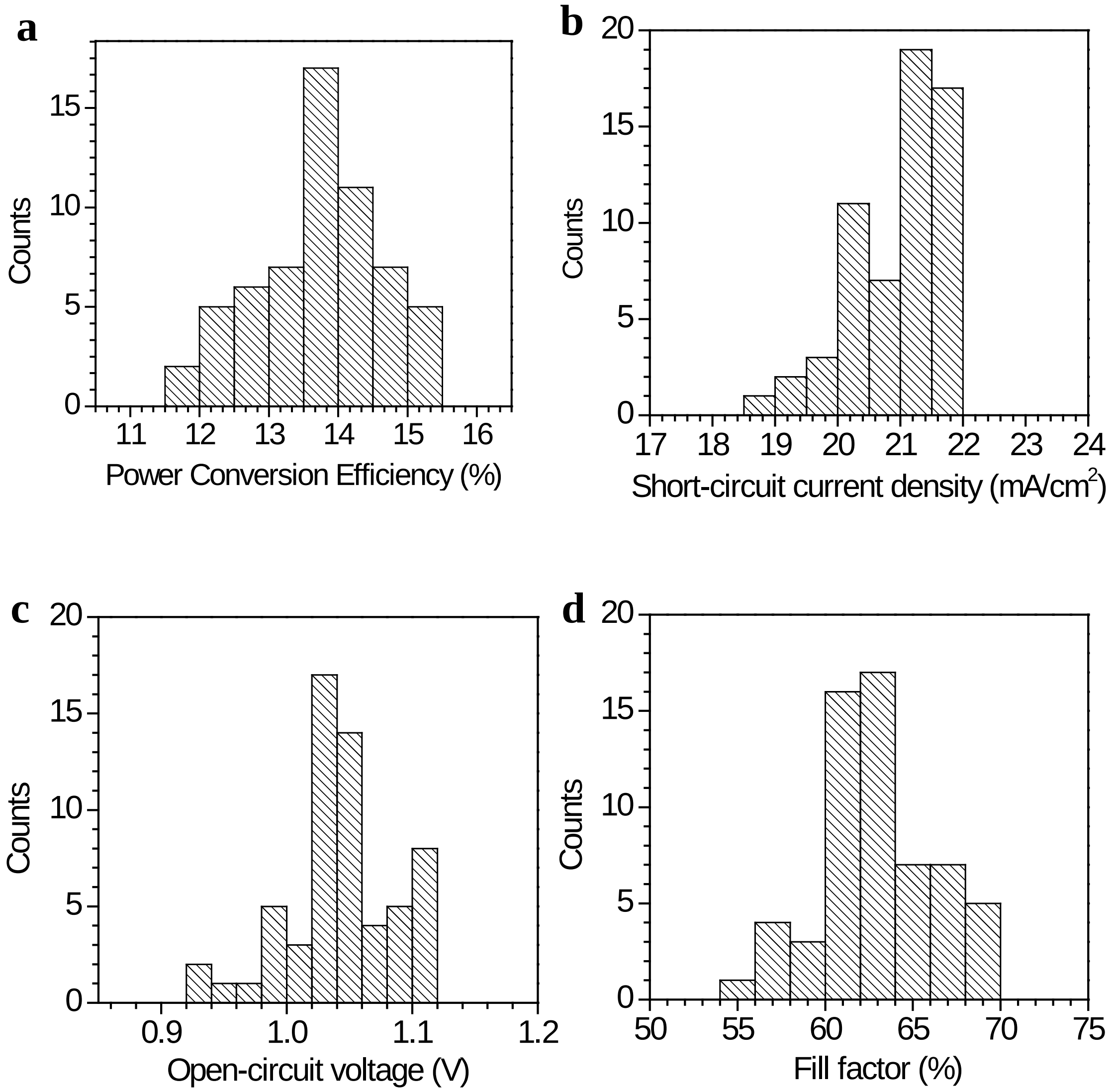

Supplementary Figure 11. Histograms of the photovoltaic parameters of flexible devices. 60 devices were measured by reverse scan with 10 mV voltage steps and 40 ms delay times under 1.5 G illumination and have 30 s of stabilization time under illumination prior to scanning. (a) Power conversion efficiency, (b) Short-circuit current density, (c) Open-circuit voltage, and (d) Fill factor.

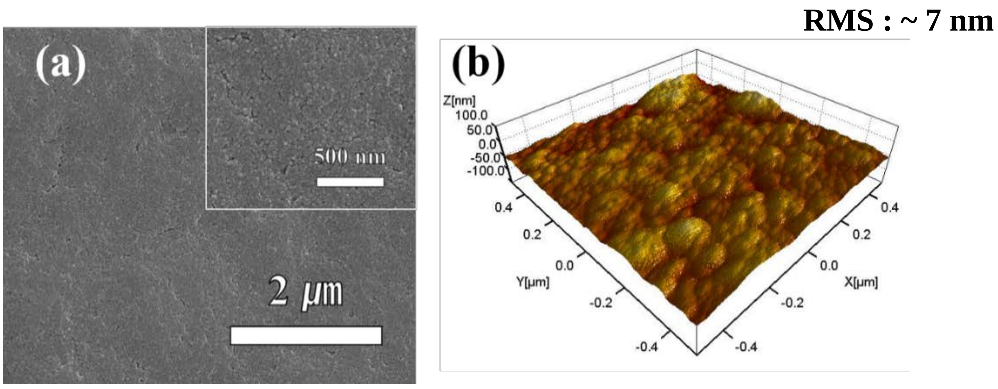

Supplementary Figure 12. Morphology of TiO_2 film. (a) Plane-view SEM and (b) AFM image of TiO_2 NPs based film.

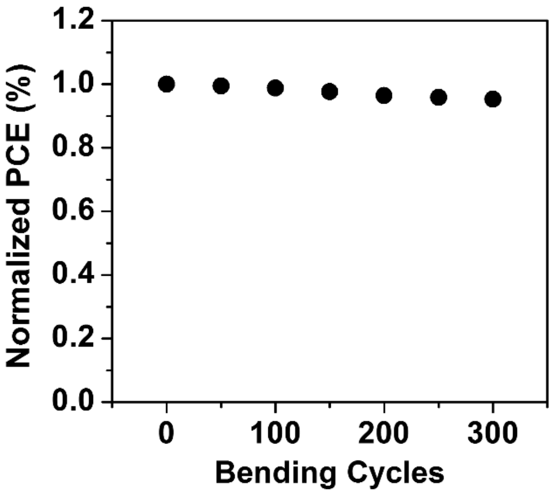

Supplementary Figure 13. Bending test. Normalized PCE of ZSO-based flexible perovskite solar cell as a function of bending cycles

Supplementary Tables

Supplementary Table 1. Photovoltaic parameters of perovskite solar cell fabricated with an ITO glass substrate.

	J_{sc} (mA/cm^2)	V_{oc} (V)	*FF* *(%)*	PCE (%)
ITO glass	22	1.03	69	15.7

Supplementary Table 2. Photovoltaic parameters of flexible perovskite solar cell.

Scan direction	J_{sc} (mA/cm^2)	V_{oc} (V)	*FF* (%)	PCE (%)
Reverse	21.6	1.06	66.8	15.3
Forward	21.4	0.99	55	11.7

Supplementary Table 3. Average photovoltaic parameters for 60 flexible devices.

	J_{sc} (mA/cm^2)	V_{oc} (V)	*FF* (%)	PCE (%)
Average parameter	20.9	1.04	62.9	13.7
Standard deviation	0.7	0.04	3.22	0.89
